# Selection index for genetic improvement of beef performance in Huaxi cattle of Xinjiang, China

**DOI:** 10.1186/s12864-026-12695-x

**Published:** 2026-02-28

**Authors:** Shah Zeb Ahmad, Ye Feng, Qian Zhang, Xubin Lu, Wenjuan Zhao, Bin Zhang, Fagang Zhong, Mengli Han, Zhi Chen

**Affiliations:** 1 College of Animal Science and Technology, Yangzhou University, Yangzhou, 225009 China; 2 State Key Laboratory for Sheep Genetic Improvement and Healthy Production, Xinjiang Academy of Agricultural and Reclamation Science, Shihezi, 832000 China

**Keywords:** Selection index, Huaxi, Body weight, Breeding, Heritability, Pedigree, Cattle

## Abstract

**Background:**

Huaxi cattle are a local breed in Xinjiang, China. This breed has gained increasing attention from farmers and breeders for its dual-purpose capabilities in both meat and dairy production. Beyond this, these animals are highly adapted to extreme environmental conditions, making them unique and valuable for livestock-enhancing programs and the food industry. To maximize economic potential, effective selection strategies should be designed to enhance production (milk and meat) and identify individuals with superior genetics, as assessed by Estimated Breeding Values (EBVs). Therefore, the current study aimed to construct selection indices for 12-month body weight (WEI-12) as a primary objective and daily milk yield (DMY) using the Smith-Hazel index method in Huaxi cattle reared in Xinjiang, China–an area that has not previously been studied.

**Methods:**

Based on estimated genetic parameters, including heritability, genetic and phenotypic variances, and covariances for the WEI-12 and DMY traits in Huaxi cattle, a selection index was calculated using the classical Smith-Hazel method. The mathematical model was fitted in RStudio (version 4.5.2, 2026) using the dplyr, tidyr, and readxl packages.

**Results:**

Nine indices (C1 to C9) were constructed by applying varying conditions, including adjustments to economic weights, selection intensity, genetic correlation, and heritability, to identify the most effective index and assess its sensitivity to selection. Among the nine indices, C1, C2, C7, C8, and C9 exhibited the highest expected genetic gains of 2.723, 2.703, 2.7152, 2.6543, and 2.9049, respectively–along with accuracy rates of 0.19, 0.18, 0.18, 0.18, and 0.19, respectively. These indices demonstrated positive genetic gain potential for WEI-12 and DMY traits under ideal conditions (e.g., high selection intensity of 10% and the original estimated genetic parameters). Furthermore, minor alterations to these ideal conditions, specifically in genetic parameters, resulted in a negligible impact on the index ’s performance. Notably, small changes were observed when genetic correlation and heritability were modified by + 10% and –20%, respectively, suggesting the index’s robustness.

**Conclusion:**

The present research established a theoretical framework utilizing pedigree based estimated genetic parameters for the construction of selection indices to increase genetic gain in 12-month body weight (primary goal) and daily milk production. This approach could be utilized by breeders in developing sustainable and productive breeding plans for Huaxi cattle. The results highlight the importance of incorporating economically relevant traits and genomic information (e.g., gBLUP) to enhance the accuracy and sustainability of breeding programs.

## Background

Huaxi Cattle are an indigenous breed found in Xinjiang Province, China. The breed has gained popularity for being both a good source of dairy and for producing high-quality meat. Because of its dual function (meat and milk), as well as its adaptability to harsh environmental conditions, Huaxi Cattle have quickly become of great interest to farmers and breeders [1]. Although increasing economic value depends on choosing an optimal strategy that properly combines bodyweight and milk yield/production, optimal selection strategies will incorporate the combined genetic correlations and heritability of traits important for breeding purposes within genetic improvement programs. By using these two fundamental genetic parameters in breeding programs, breeders have tools to predict the benefit of a selection as well as understand the genetic composition of economically important traits so that they can make better-informed selections based on evidence from genetic data collected about the respective traits [2, 3].

The genetic correlation between body weight and milk production in Huaxi cattle is critical for developing an appropriate and balanced breeding plan. A Selection Index will help ensure that an accurate evaluation of an animals overall genetic value is made, by combining many traits into a single score that can be easily used by both the breeder and the producer. These selection index methods include the Smith-Hazel Index, a classical linear selection index that weighs traits according to their economic values and genetic parameters [4, 5]. Conversely, the Restricted Selection Index imposes constraints on specific traits to prevent undesirable changes while simultaneously improving others. The most sophisticated and precise method of measurement for Selection Index coefficients is the BLUP/index method since this method allows for the use of genomics in conjunction with traditional pedigree information. This methodology is particularly useful for those who are developing dual-purpose breeds (example: Huaxi Cattle) as both production and growth traits need to be evaluated [6]. To create indices of economic importance, genetic parameters must be estimated. To create indices of economic importance, genetic parameters must be estimated, allowing for the accurate assignment of economic weights – determined by production costs and market demand–for each trait included in the index. Therefore, when selection is based on these indices, it is much more consistent with the producer’s economic conditions, thus increasing the practical value of genetic improvement programs [7, 8].

The estimation of genetic parameters and economic weights of traits of interest must be correct to develop a selection index for Huaxi cattle. It is a procedure that uses a large amount of phenotypic and pedigree data to infer heritability, genetic correlations, and breeding values [9]. In addition, economic modeling is needed to establish the relative value of each trait in the overall breeding objective [10]. For this purpose, this study aimed to provide a theoretical framework of a selection index for 12-month weight (WEI-12) as a primary objective and Daily Milk Yield (DMY) based on the estimated genetic parameters [11, 12] using the Smith-Hazel Index method described by Yamada, Yokouchi [4]. This framework could be utilized by breeders and farmers to inform animal selection, ultimately leading to genetic enhancement and economic rationality in Huaxi cattle production.

## Materials and methods

### Data collection

Data were collected from a cattle farm in Xinjiang Province, China, encompassing 2992 cows (2020–2024). The original dataset included milk traits, specifically 305-day total milk yield (MY), milk fat percentage (MF%), and milk protein percentage (MP%). Weight-related data recorded individual and phenotypic information, such as birth season, parity, withers height, body weight, chest girth, body length, height at the cross, cannon bone girth, and abdominal girth. Pedigree data were provided by the Xinjiang Agricultural University from the Huaxi Cattle Farm.

Researchers traced the pedigree of each Huaxi cow with phenotypic records, achieving an average tracing depth of five generations to ensure the completeness and accuracy of the pedigree information. A total of 3891 Huaxi cattle were used, consisting of 296 bulls and 3595 cows, for genetic analysis. Tailored Total Mixed Rations (TMR) formulations (Table 1) enhance production performance and animal health.

**Table 1:** Formulation of feed, including a total mixed ration (TMR), for specific age stages such as calf, juvenile, adult lactating, and dry cow

Life stage	Feed formulation*	Nutritional value of the feed
Calf stage	corn (40–45%) + corn silage (25–30%) + soybean meal (15–18%) + mineral premix (2%)	11.812.2 MJ /kg of metabolizable energy, 18.020.5% of crude protein, 2832% of neutral detergent fiber, 0.700.85% of calcium, 0.400.48% of phosphorus and 800010,000 IU/kg of vitamin A
Juvenile stage	be corn (50–55%) + corn silage (30–35%) + rapeseed meal (12–15%), + dicalcium phosphate (1.5%) + salt (0.5%)	12.2128 MJ/kg metabolizable energy, 16.0178% crude protein, 2024% acid detergent fiber, 0.80095% calcium, and 0.45052% phosphorus.
Adult lactating phase	corn (4550%), whole plant silage (4045%), soybean meal (1012%), corn gluten meal (58%), calcium carbonate (1.2%), and bentonite (1.0%)	12.5–13.2 MJ/kg of metabolizable energy, 17.5–19.2% of crude protein, 0.60–0.70% of rumen degradable protein, 0.90–1.15% of calcium, 0.48–0.58% of phosphorus and 0.28–0.32% of magnesium
Dry cow stage	corn (3540%), oat hay (2025%), corn silage (2530%), cottonseed meal (810%), and premix (2.5%)	10.210.8 MJ/kg of metabolizable energy, 12.5–14.0% crude protein, 0.50–0.62% of calcium and 0.30–0.38% of phosphorus

* Adjustments to the diet for high-yielding cows involved increasing the percentage of concentrate by 1520%, replacing 30% cottonseed meal with soybean meal, and introducing rumen-protected fat at 35%. The resulting nutritional characteristics were: 13.0 MJ/kg metabolizable energy, 18.5% crude protein, 5.5–6.5% fat content, and 1.05–1.25% calcium

The said farm has a standardized farming system for breeding practices having access of proper clean water as well as a proper and well-formulated diet or Total Mixed Ration (TMR) for the animals. TMR is a special type of feed having balanced nutrient contents formulated under standardized processing i.e. its mixing protocol strictly adheres to animal nutritional requirement models to ensure the nutritional need for the animals at their specific production stage as shown in Table 1.

### Estimation of genetic parameters

To calculate the selection indices for WEI-12 and DMY, the genetic parameters analyzed by Feng, Han [11] Feng, Zhao [12] through blupADC package [13] in RStudio (version 4.4; 2024) [14] were employed. Their analysis included descriptive statistics, genetic parameters such as heritability, heritability standard error (h^2^ SE), additive genetic variance (V^2^), additive genetic variance standard error (V^2^ SE), and a correlation study examining phenotypic and genotypic correlations for milk and body weight.

### Selection index

The selection index coefficient was calculated using the Smith-Hazel Index, as described by Yamada and Yokouchi [4]. The model was fitted in RStudio (version 4.5.2, 2026) using dplyr, tidyr, and readxl packages. The selection index theory is explained below.

To change the means of m traits by the amount of *Q*_*j*_, *j* = 1, 2, …, *m*. Then, *Q* which is defined as an *m x 1* vector of intended genetic changes for m traits assigned by the breeder is,

1 $$\:{Q}^{{\prime\:}}=[{Q}_{1},\:{Q}_{2\:},\dots\:..\:{Q}_{m\:}]$$

2 $$\:I=b{\prime\:}X$$

Where *X* is a vector of information (*n* × 1), primarily phenotypic measurements for selection, *and b* is the weighting factor for breeder objectives (*n* × 1).

The expected genetic gain per generation is defined as:

3 $$\:\varDelta\:{G}_{j.\:I}=\:\frac{{i}_{I}}{{\sigma\:}_{I}}Cov\left({G}_{j,\:\:}I\right)$$

Where:

$\:{i}_{I}$ is standardized selection differential on the index, $\:{\sigma\:}_{I}$ is the standard deviation of the index and $\:Cov\left({G}_{j,\:\:}I\right)$ is the covariance of the breeding value of the *jth* trait and the index. The covariance can be rewritten as;

$$\:Cov\left({G}_{j,\:\:}I\right)\:=\:Cov\:\left({G}_{j,\:\:}\:b'X\right)$$

*= [G’Rb]*_*j*_ (4a).

Where *[G’Rb]*_*j*_ is the jth element of *[G’Rb].*

Substituting Eq. (4a) into (3), we obtain:

4b $$\:\varDelta\:{G}_{j.\:I}=\:\frac{{i}_{I}}{{\sigma\:}_{I}}{\left[G'Rb\right]}_{j}$$

Now, the *m x 1* vector representing expected genetic gain per generation across *m* traits denoted by *∆G*, is shown below:

4c $$\:\varDelta\:G=\:\frac{{i}_{I}}{{\sigma\:}_{I}}\left[G'Rb\right]$$

Where:

*G* is an *n × m* matrix representing genetic covariances between elements of *X* and those in *Q*, on an individual basis. *R* is an *n × n* diagonal matrix of *r*, Wright’s coefficient of relationship between the candidate and the relatives who provide information for *X*_*i*_.

For four generations of selection, assuming unchanged population parameters, the vector Q is:

5a $$\:Q=q\varDelta\:G$$

5b $$\:=q\left(\frac{{i}_{I}}{{\sigma\:}_{I}}\right)\left[G'Rb\right]$$

Regarding *b*, Eq. (5b) becomes:

$$\:q\left(\frac{{i}_{I}}{{\sigma\:}_{I}}\right)$$ *= 1* (6).

hence,

7 $$\:b\:=\:{\left(G{\prime\:}R\right)}^{-1}Q$$

The standard deviation of $\:{\sigma\:}_{I}$ is,

8 $$\:{\sigma\:}_{I}=\:\sqrt{b{\prime\:}Pb}$$

where *P* is an *n × n* matrix of covariances between elements of *X*.

For genetic gain, the following equation was considered:

9 $$\:\varDelta\:G*=\:\frac{{i}_{I}}{{\sigma\:}_{I}}\left[G*'Rb\right]$$

Where $\:\varDelta\:G*$ is the vector, whose elements are consisted of $\:\varDelta\:G$_k. I_, *k* = 1, 2, …, m, l, and. G* is an *n x 1* matrix of genetic covariance (elements σ_Gtik_). After 4 generations of selection, the total changes of these traits are,

10 $$\:Q*=q\varDelta\:G*\:=\:G*'Rb$$

Hence Eq. (7) becomes,

11 $$\:b={{P}^{-1}RG\left[G{\prime\:}R{P}^{-1}RG\right]}^{-1}Q$$

### Calculation of selection index accuracy

The accuracy of the selection index was calculated using the following mathematical model.

$$\:rHI\:=\:\sqrt{\frac{bTPb}{aTGa}}$$

Where:

*rHI* = accuracy of selection index.

*b* = vector of index weights.

*P* = phenotypic covariance.

*a* = vector of economic weights.

*G* = genetic covariance matrix.

*T =* transpose (flip rows and columns).

### Sensitivity of the selection index

Nine distinct conditions were constructed by adjusting parameters including selection intensity, economic weights, heritability, and genetic correlation. For example, in condition C1, a standard selection intensity of 10% was employed, with the remaining parameters held constant. Condition C2 utilized a 20% selection intensity, alongside economic weights of 0.5 and 1 for WEI-12 and DMY, respectively. Conditions C3 and C4 involved variations in selection intensity, coupled with economic weights for the traits that were opposite to those in C1 and C2 (1 and 0.5 for WEI-12 and DMY, respectively). Conditions C5 and C6 were designed to maintain all parameters constant while setting the economic weight to 1 for both traits. In conditions C7 and C8, the original heritability was altered by ± 10% to determine selection index coefficients. Finally, condition C9 decreased the genetic correlation between WEI-12 and DMY to -20% to obtain the index coefficient.

## Results

Based on published data [11, 12], we constructed a selection index for genetic improvement, with primary objectives of 12-month weight (WEI-12) and daily milk yield (DMY). The estimated genetic parameters for WEI-12 and DMY, derived from the studies of Feng and Han [12] and Feng and Zhao [11], are listed in Table 2. The calculated genetic correlation coefficient and covariance between WEI-12 and DMY are − 0.15 and − 0.75, respectively.

**Table 2:** Phenotypic and genetic variances, and heritability of WEI-12 and DMY in Huaxi cattle

Trait	No. observations	Mean	SD	σ^2^_P	V^2^_A	h^2^
WEI-12(Kg)	2992	357.15	28.1	789.61	11.21	0.39
DMY(Kg)	2992	28.50	5.9	34.81	2.25	0.39

σ^2^_*P* Phenotypic variance, V^2^_*A* Genetic variance, *h*^2^ Heritability, *SD* Standard deviation

### Selection index

Table 3 presents selection indices designed to achieve genetic improvement in 12-month body weight (WEI-12) as a primary objective and Daily Milk Yield (DMY) in Huaxi cattle from Xinjiang, China. Applying various conditions i.e. adjusting selection intensity, economic weights, genetic correlation and heritability of the traits, nine types of index coefficients were obtained i.e. C1 to C8, in which C1, C2, C7, C8 and C9 indices are the most effective with a cumulative expected genetic gain after four generations equal to 2.723, 2.703, 2.7152, 2.6543 and 2.9049 having accuracy rate of 0.19, 0.18, 0.18 and 0.18 respectively. The results also show that higher selection intensity leads to higher expected genetic gain. The study confirmed that the total genetic gain for both traits was cumulatively higher than for either trait individually.

**Table 3:** Selection index coefficient (b) and their accuracy (r_HI) with respect to selection intensity, economic weights, genetic correlation and Heritability adjustment

Condition	Trait	Selection intensity	Economic weight	h^2^	r_A_	b	ΔG^*^		r_HI
							**Per generation**	**After 4 generations**	
C1	WEI-12	10%	0.5	No change	No change	0.008	0.1083	0.4331	0.19
	DMY		1.0			0.05	0.0381	0.1525	
	Cumulative						0.6807	2.723	
C2	WEI-12	20%	0.5	No change	No change	0.008	0.0304	0.4331	0.18
	DMY		1.0			0.05	0.0864	0.1525	
	Cumulative						0.543	2.703	
C3	WEI-12	10%	1	No change	No change	0.014	0.0808	0.3231	0.13
	DMY		0.5			0.030	0.035	0.1398	
	Cumulative						0.7686	3.0744	
C4	WEI-12	20%	1	No change	No change	0.014	0.0643	0.257	0.13
	DMY		0.5			0.0203	0.0278	0.1112	
	Cumulative						0.6114	2.4456	
C5	WEI-12	10%	1	No change	No change	0.014	0.0643	0.2571	0.14
	DMY		1			0.053	0.0554	0.2217	
	Cumulative						0.851	3.4039	
C6	WEI-12	20%	1	No change	No change	0.014	0.0514	0.2057	0.14
	DMY		1			0.053	0.0443	0.1774	
	Cumulative						0.6808	2.7232	
C7	WEI-12	10%	0.5	+ 10%	No change	0.008	0.038	0.1521	0.18
	DMY		1.0			0.05	0.108	0.4319	
	Cumulative						0.6788	2.7152	
C8	WEI-12	10%	0.5	-10%	No change	0.008	0.038	0.1521	0.18
	DMY		1.0			0.05	0.108	0.4319	
	Cumulative						0.6788	2.6543	
C9	WEI-12	10%	0.5	No change	-20%	0.007	0.0604	0.2416	0.19
	DMY		1.0			0.064	0.1134	0.4536	
	Cumulative						0.7015	2.9049	

Heritability (h^2^), genetic correlation (r_A_), index coefficient (b), expected genetic gain (ΔG), and accuracy (r_HI)

^*^The genetic gain is in kilogram (Kg)

The results also demonstrate the sensitivity of indices, including selection intensity, economic weights, and variation in heritability, for the traits. For example, with a selection intensity of 10% and economic weights for WEI-12 and DMY equal to 0.5 and 1.0, respectively, the genetic gain was high, reaching 0.6807 per generation and 2.723 units after four generations. However, when the selection intensity increased to 20%, the accuracy rate decreased slightly in C2 to 0.18, without a corresponding change in the expected genetic gain.

## Discussion

This study has focused the estimated genetic parameters from a dataset of 2992 Huaxi cattle for body weight and milk traits. All traits exhibited high heritability and repeatability, accompanied by significant genetic correlations ranging from–1 to + 1 [15]. This large data set provides sufficient information for subsequent genetic evaluation and analysis, enhancing the accuracy and credibility of the research outcomes. Compared to Huaxi cattle, Luxi Yellow cattle demonstrated slightly lower heritability (0.20–0.33), while Jinnan cattle exhibited a wider variation (0.18–0.36). However, it is crucial to acknowledge that persistent inbreeding can lead to a loss of population genetic diversity, consequently increasing the susceptibility of animals to biological threats, such as new pathogens and environmental pressures [16, 17]. Furthermore, inbreeding is rare in Huaxi cattle (dual-purpose cattle), which is likely to be beneficial for production within the dairy industry [1]. The study also quantified the degree of covariation among traits attributable to genetic factors, including additive genetic effects–an important aspect for understanding genetic associations and gene pleiotropy when assessing breeding practices [15, 18]. These parameters were analyzed to construct selection indices for various traits in Huaxi cows.

This study presents the first attempt to construct selection indices for genetic improvement of 12-month body weight (WEI-12) and daily milk yield (DMY) traits in Huaxi cattle from Xinjiang, China. A total of nine selection indices were constructed keeping different conditions such as changing the economic weights of the traits, adjusting selection intensity and variation in heritability of the traits to get more suitable index coefficient as well as to test their sensitivity and robustness. The most effective indices identified were C1, C2, C7, C8, and C9, achieving cumulative genetic gains of 2.723, 2.703, 2.7152, 2.6543, and 2.9049 units, respectively, with moderate accuracy rates of 0.19, 0.18, 0.18, 0.18, and 0.19. Furthermore, the results confirmed that increased selection intensity correlates with higher expected genetic gain, indicating that selecting a smaller, more genetically superior population will accelerate progress per generation. Similarly, when heritability increased by 10% (in C7), the genetic gain increased slightly to 2.715, compared to a 10% decrease (in C8), which resulted in a genetic gain of 2.6543. Similarly, when genetic correlation was changed to -20% a very small amount of change was observed in the expected genetic gain with a moderate accuracy of 0.19. The slight change in genetic gain and accuracy shows the robustness of the five indices i.e. C1, C2, C7, C8 and C9. The current study also confirmed that the total genetic gain is high while taking both of the traits cumulatively than the individual traits demonstrating the benefits for multi-trait selection.

Although the genetic correlation between body weight and milk yield is –0.15, consistent with existing literature such as the study of Canaza-Cayo and Lopes [19], it was clarified that genetic correlations between mature body weight and 305-day milk yield ranged from –0.19 ± 0.04 in Brazilian Girolando cattle. Similarly, Lee and Pollak [20] concluded that the genetic correlation between body weight and milk yield was negative, ranging from –0.08 to –0.16 for birth weight, − 0.04 to –0.21 for weaning weight, and –0.12 to –0.19 for yearling weight. Wang and Li [21] reported similar results. However, the negative correlation observed in this study between WEI-12 and DMY in Huaxi cattle from Xinjiang provides a basis for further genetic investigation. Despite the antagonistic pattern of genetic correlation between these traits, the primary objective for selection index construction was the genetic improvement of beef performance. But taking milk yield alongside is because the cumulative genetic gain is higher than individual genetic gain. Taken to account the work of Lin [5], it can be stated as “same selection is not the same always" means that the same selection index almost always been used throughout a given selection experiment. Inevitably, this could lead to misleading results and interpretation. Therefore, a positive genetic gain may be expected even if heritability and correlation are not significant. Accounting for this limitation, and the concept of within-family Mendelian sampling variation, this approach was extended to four generations, allowing breeders to assess trait performance in an intermediate generation, which will be considered. The accuracy of this theoretical framework will be further increased by performing gBLUP, a limitation of the present study that is under consideration for future research.

A key limitation of this study lies in the construction of index coefficients for WEI-12 and DMY traits, core production characteristics in Huaxi cattle, while neglecting other economically relevant traits such as birth weight, fertility, and feed efficiency. Initially, research indicates that Huaxi cattle exhibit accelerated weight gain during early growth stages and demonstrate higher heritability estimates [5, 22]. Secondly, this approach is a clear methodological framework and does not reflect multi-trait selection model essential in contemporary breeding. As emphasized recently in the literature that it is critical to improve feed efficiency while reducing environmental footprint [23]. Focusing solely on production traits can negatively affect other functional traits, including animal health and fertility [24]. Consequently, incorporating resilience to specific climatic conditions is crucial [25, 26]. Future research should prioritize a multi-trait selection framework and the derivation of economic weights for all relevant economic traits–including efficiency, health, and emissions–to determine optimal resource allocation that maximizes aggregate genetic gain, ensuring long-term resilience and sustainability of the population.

Many genes and their polymorphisms, such as single nucleotide polymorphisms (SNPs), have been identified as exerting direct and indirect effects on growth and fattening in various cattle breeds. Specifically, genes including oxidized low-density lipoprotein receptor 1 (*OLR1*), lactoferrin (*LTF*), stearoyl-CoA desaturase (*SCD*), beta-lactoglobulin (*LGB*), thyroglobulin (*TG*), annexin A9 (*ANXA9*), myogenic factor 5 (*MYF5*), protein kinase AMP-activated non-catalytic subunit gamma 3 (*PRKAG3*), and pituitary-specific transcriptional factor 1 (*PIT1*), have been associated with fattening performance in Simmental cattle [27]. Furthermore, a multi-trait genome-wide association study (M-GWAS) by Forutan and Ross [28] identified genes related to height in cattle, including *IRAK3*, *HELB*, *HMGA2*, *LAP3*,* FAM184B*,* LCORL*, *PPM1K*, *ABCG2*, *MED28*, *PLAG1*, *BPNT2*, *UBXN2B*,* CTNNA2*, *SNRPN*, and *SNURF.* Moreover, some of the candidate genes and their SNPs are identified in associations which drive the various traits in Holstein–Friesian purebred. These associations among the SNPs are *LEP* × *GHR*, *IGF1* × *LEP*, *FABP4* 3691 × *FABP4* 2834, and *FAP4* 3533 × *LEP* interactions [29]. These findings provide a valuable references for future research utilizing genome-wide association studies (GWAS) on Huaxi cattle to develop selection programs for sustainable cattle production.

Studies have considered selection strategies to improve genetic gain for various traits, including selection for reduced milk traits, crossbreeding, and gene editing, as well as selection for physiological and cellular traits and selection for high immune response [30–33]. For instance, milk yield in combination with heat stress resistance in hot regions of the world has been used to improve heat stress tolerance and increase milk yield in areas experiencing increased heat waves due to climatic change [34]. This highlights a key concept in modern dairy breeding: the economic value of milk is intrinsically linked to its components. Fat and protein content are usually the key characteristics that are rewarded by modern payment systems. The undesirable dilution of these solids can be caused by choosing to yield alone, negatively correlated, which has been well-established [35]. By including sufficient economic weight for composition and ensuring Even Genetic Advancement, the index employed here is ideal because it facilitates Balanced Genetic Advancement. In addition to being consistent with worldwide Dairy Cattle Breeding Trends, Selection Indices, including the Net Merit Index (NMI) used in the USA, have been instrumental in simultaneously improving Yield, Reproductive Efficiency, and all Health Components of a Cow [36]. We examine joint selection in Huaxi Cattle, with the goal of increasing the genetic gain for DMY and WEI-12 in the Xinjiang Region of China over four generations. This is the first study that has done this. Although some research has developed optimal models for milk production and other traits (e.g., heat stress) in other breeds like Brazilian Holsteins, the same has not been done for Huaxi Cattle [37].

During index construction, we account for traits such as birth weight, which remain unchanged, as high birth weight is associated with an increased risk of dystocia and may not align with the preferred mature size of cows in terms of optimal control and maintenance expenses [38, 39]. A multi-stage growth index can enable breeders to select animals with an overall growth curve exhibiting a more optimal trajectory, leading to improved efficiency and reduced adverse outcomes. This reflects the sophisticated application of random regression models in genetic assessment, which provides a more accurate representation of growth curves throughout an animal’s lifespan [40].

The simultaneous use of multiple indices for production and growth facilitates an advanced and specialized breeding program. This practice is central to contemporary animal breeding, with the goal of maximizing an aggregate breeding value that integrates all economically significant traits [41]. For the Huaxi herd, this translates to utilizing females with high WEI-12 indices as donors, who are then bred to produce the next generation of animals mated with elite sires to achieve high growth performance. High-growth index females can also be incorporated into terminal crossing systems to enhance beef production or improve the structural development of replacement heifers. This approach is valuable for managing genetic trade-offs and mitigating the unintended consequences of single-trait selection. The Smith-Hazel Index method, as described by Yamada and Yokouchi [4], has demonstrated improved performance through the application of genomic technologies, representing an efficient and sustainable method of genetic improvement.

Apart from the results provided by this study, several limitations warrant consideration in future research to enhance this approach. These limitations include the lack of genomic data; the study employs a pedigree-based quantitative genetic approach without incorporating genomic analysis, such as gBLUP. This restricts the study’s significance, as reported by VanRaden et al. (2009) [42], which demonstrated that gBLUP analysis increased breeding value accuracies by 20–50%. Similarly, Harris B, Johnson D and Spelman R (2009) [43] reported that the reliabilities of genomic predictions were 16–33% higher than the breeding values that were purely based on parental information. As previously discussed, cumulative genetic gain exceeds individual genetic gain, and accurate genotype imputation enhances this pattern and the reliability of estimated breeding values, which is crucial for maximizing cumulative gains in complex traits. Considering imputation accuracy as outlined by Júnior and Costilla (2025) [44], incorporating this into the current approach could facilitate the construction of more effective selection indices for antagonistic traits, such as WEI-12 and DMY. Furthermore, the lack of genomic studies limits the assessment of linkage disequilibrium within the selected Huaxi cattle population. Finally, the index derived for Huaxi cattle reared in the Xinjiang region of China may have limited generalizability to other regions with distinct climates.

## Conclusion

Pedigree-based genetic parameters and the Smith-Hazel Index method were employed to construct an index for the genetic improvement of 12-month body weight (WEI-12) as a primary objective and daily milk yield (DMY) in Huaxi cattle from Xinjiang, China. Analysis of the data resulted in the creation of nine indices, with five identified as the most robust. These indices (C1, C2, C7, C8 and C9) generated high expected cumulative genetic gain with moderate accuracy. The performance of these indices was insensitive to variations in selecting intensity, heritability and genetic correlation. The combined selection method yielded higher cumulative genetic gain than either trait selected individually, despite the negative genetic relationship between WEI-12 and DMY. Consequently, utilizing multiple traits in selection increases the potential for genetic improvement. The findings of this study highlight the importance of incorporating economically important traits and genomic information (e.g., gBLUP) to enhance selection accuracy and breeding sustainability. This framework can be utilized by breeders to develop and improve dual-purpose breeding programs tailored to the climate of Xinjiang province.

## Data Availability

This study used previously published pedigree-based estimated genetic parameters (see the Methods section for references) to construct a selection index. Furthermore, the script used for data analysis is available at [https://github.com/shahzebahmad45/Script.git].

## Abbreviations

WEI-12: 12-months weight in kilogram
DMY: Daily milk yield
TMR: Total Mixed Ration
NM$: Net Merit Index
C1, C2…C9: Condition 1, Condition 2 … Condition 9 are the various conditions in which the parameters were changed to construct and check the sensitivity of selection index coefficients.
